# Reliability of muscle stiffness measures in popliteus, medial and lateral gastrocnemius muscles by ultrasound shear wave elastography in participants with knee osteoarthritis accompanied by myofascial trigger points

**DOI:** 10.1186/s12891-024-07351-y

**Published:** 2024-03-19

**Authors:** Mohsen Shams, Noureddin Karimi, Mohsen Vahedi, Peyman Kamali Hakim, Fahimeh Zeinalkhani, Leila Rahnama

**Affiliations:** 1 Department of Physiotherapy, University of Social Welfare and Rehabilitation Sciences, Tehran, Iran; 2 Department of Biostatistics and Epidemiology, Substance Abuse and Dependence Research Center, University of Social Welfare and Rehabilitation Sciences, Tehran, Iran; 3 Department of Radiology, Tehran University of Medical Sciences, Imam Khomeini Hospital, Tehran, Iran; 4 Rongxiang Xu School of Health and Human Services, California State University, Los Angeles, Los Angeles, CA USA

**Keywords:** Knee osteoarthritis, Stiffness, Ultrasonography, Elastography, Trigger point, Gastrocnemius, Popliteus

## Abstract

**Background:**

The objective of this investigation is to evaluate the consistency of intra-rater and inter-rater assessments utilizing ultrasound elastography to examine the muscle stiffness of the popliteus and gastrocnemius (medial and lateral heads) in patients with knee osteoarthritis accompanied by myofascial trigger points.

**Methods:**

Thirty individuals with knee osteoarthritis accompanied by myofascial trigger points were assessed. Two examiners independently measured the muscle stiffness levels of the popliteus and gastrocnemius (medial and lateral heads) three times using ultrasound elastography in the first session. The second session was conducted one week later.

**Results:**

In the initial test session, the mean shear modulus values for the popliteus and gastrocnemius (medial and lateral heads) muscles were measured as follows for tester 1 (12.75, 13.72, 14.13 kPa) and tester 2 (11.66, 12.81, 13.17 kPa). During the retest session, the previously measured variables by tester 1 and tester 2 yielded the following values: (12.61, 13.43, 14.26 kPa) and (11.62, 12.87, 13.30 kPa) respectively." Good to excellent intra-rater reliability (ICC = 0.912—0.986) and inter-rater reliability (ICC = 0.766—0.956) were reported for the shear moduli of the popliteus, medial and lateral gastrocnemius muscles.

**Conclusions:**

The assessment of muscle stiffness in the popliteus and gastrocnemius (medial and lateral heads) using ultrasound elastography is a reliable method in patients with knee osteoarthritis accompanied by myofascial trigger points.

## Background

Knee osteoarthritis is one of the most common rheumatological and musculoskeletal disorders. Currently, the prevalence of knee osteoarthritis in Europe varies between 2 and 17%. The number of affected individuals and their age are increasing, leading to a significant economic and social burden on the world population [1]. Knee osteoarthritis is a chronic disease associating with the degeneration of articular cartilage and surrounding soft tissues of the joint. This condition causes pain, rigidity, limited range of movement, reduced physical performance and disability [2–4]. It has been shown that in osteoarthritis, there is a relationship between the existence of myofascial trigger points within the muscles related to knee joint in knee osteoarthritis and pain and functional restrictions [5]. The prevalence of myofascial trigger points in the muscles around the knee in knee osteoarthritis patients is reported to be 11% to 50% [6]. The myofascial trigger points present in the lower limb muscles can be a source of individualized pain and joint stiffness in individuals with knee osteoarthritis [3, 7].

Trigger points are hyperirritable nodules in taut bands of skeletal muscle, and based on their clinical activity, they can be classified into active and latent types [1]. Active trigger points may contribute to general movement dysfunction (such as stiffness and limited range of motion). Latent trigger points, although not inherently painful, are prevalent clinical findings in symptomatic and asymptomatic individuals that can also lead to general movement dysfunction [8].

Objective measurement of tissue stiffness in a clinical setting is essential for individuals whose treatment goal may involve reducing muscular stiffness (such as tissue massage, dry needling, etc.) [9]. Additionally, objective measurement of stiffness can be useful for monitoring treatment effectiveness. Shear wave elastography (SWE), a relatively new approach, is used to objectively measure tissue stiffness in muscles [9, 10].

In SWE, Young's modulus is estimated as an indicator of stiffness based on the shear wave propagation velocity of ultrasound. Previous studies have shown that SWE measurements are reliable across a wide range of muscles. Since the measured muscle shear modulus using SWE is linearly related to Young's modulus, this technique is used to provide a localized estimation of muscle stiffness. Previous studies have shown that SWE measurements are reliable across a wide range of muscles [10], However, there has been no research on the popliteus muscle (PM), medial gastrocnemius (GM) and lateral gastrocnemius (GL) muscles in the subjects studied in the present research. The objective of this investigation is to evaluate the consistency of intra-rater and inter-rater assessments utilizing ultrasound elastography to examine the muscle stiffness of the PM, GM and GL in patients with knee osteoarthritis accompanied by myofascial trigger points.

## Methods

### Study participants

Individuals with knee osteoarthritis were selected and included in the study if they had myofascial trigger points in the gastrocnemius and popliteus muscles unilaterally or bilaterally, and fulfilled the criteria for inclusion and exclusion. The criteria for inclusion encompassed the subsequent factors: the existence of a minimum of one latent or active trigger points in the gastrocnemius and popliteus muscles, an age range 50—70 years, and a duration of pain exceeding 3 months [1, 11]. The exclusion criteria were as follows: fibromyalgia [1], severe knee pain and inflammation, total knee replacement on the affected side [12, 13], any fracture or Previous lower limb surgery [11, 12] and intense sports training [14].

### Clinical assessment

The standard criterion for the diagnosis of knee osteoarthritis was the grade I-III based on the Kellgren and Lawrence scale in X-ray [6, 11]. The standard criterion for diagnosing the presence of trigger points was the presence of at least one trigger point in the mentioned muscles, which is defined as a taut band that is palpable and elicits a painful response upon touch [15]. The examiners marked the key trigger points in the central area of the muscles belly for measurement.

### Muscle stiffness measurement

Muscle stiffness was assessed utilizing SWE with a linear probe (2–10 MHz, Super Sonic Imagine, Aixplorer, Germany). The selection of the imaging site was based on previous established studies [16, 17]. It is believed that the most accurate zone for conducting SWE assessment locates within the thickest part of the muscles. We selected only one circle inside each ROI to assess the muscle stiffness. However, the circle dimension was 5 mm which was adequate to represent the inherent stiffness of gastrocnemius. The measurement site for the PM was determined as the point where two lines intersect: the thigh midline and the line connecting the lateral epicondyle of the femur to the rear surface of the upper tibia [14]. The measurement location for the MG and LG muscles was defined as 30% of the proximal calf length [18, 19]. The length of the MG was determined from the inner part of the transverse crease of the popliteal fossa to the highest point of the lateral malleolus. For the LG, leg length is measured from the lateral part of the transverse crease of the popliteal fossa to the highest point of the medial malleolus. The length was measured using a measuring tape [18]. A black oil-based pen was used for marking the measurement site. We detected the central portion of the popliteus muscle employing B-mode ultrasound. The linear probe was aligned in parallel with the fascicles of the PM, MG, and LG muscles and perpendicular to the skin over the marked area [20].

**Fig. 1:**
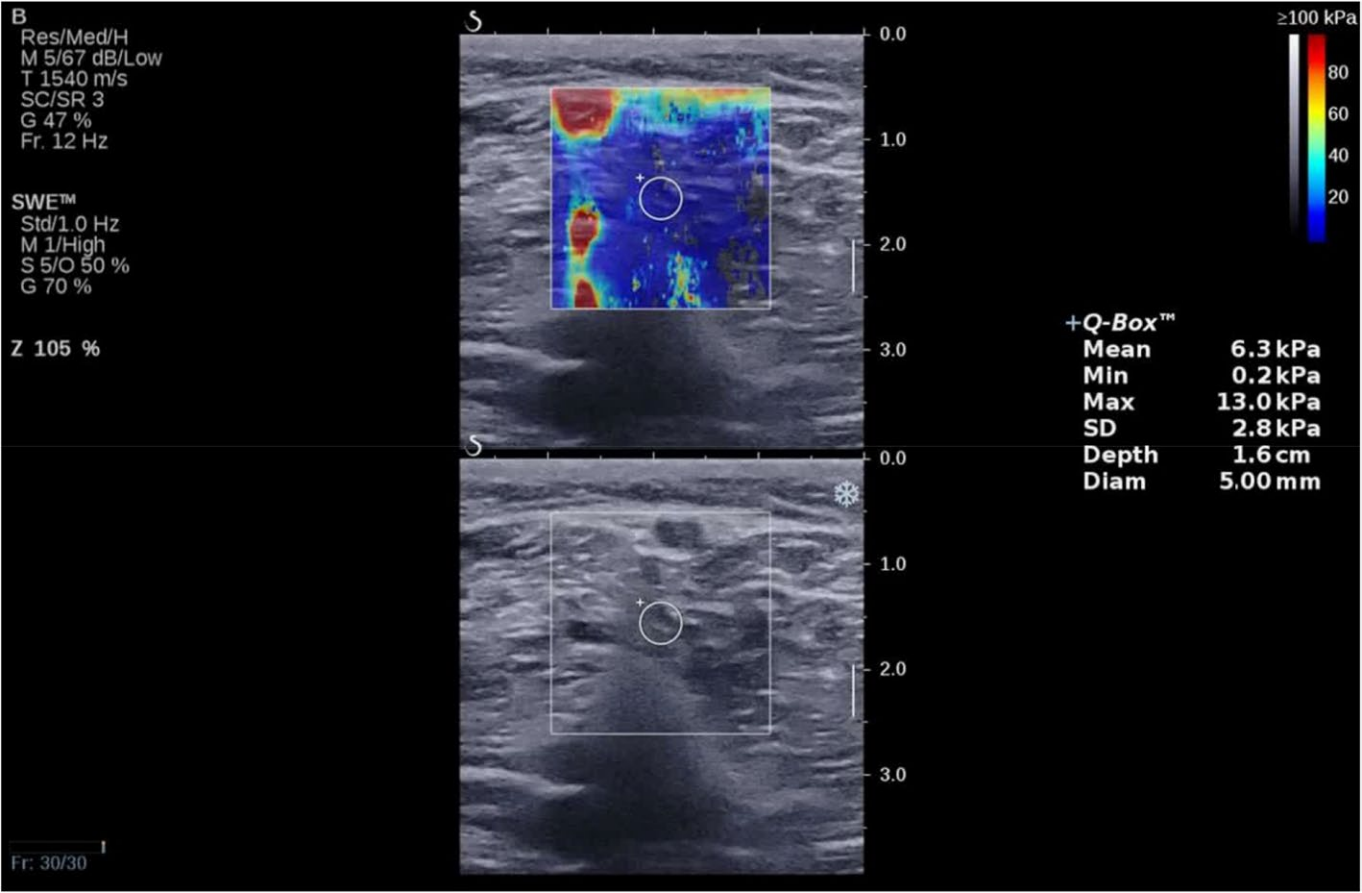
Typical elastography maps of the popliteus muscles. The upper images display color-coded boxes representing muscle stiffness. The bottom images show grayscale sonograms of the muscle. The measurement of shear modulus is conducted within a circular area, and the Q-Box is displayed on the right side for reference

Muscle stiffness was measured at this location for a duration of 5 s, following visual confirmation [14, 20]. For each measurement, a square region of interest (ROI) with dimensions of [20 × 20 mm] was set near the center of the SWE image, where the muscle appeared thickest. Additionally, for quantitative analysis, a circle with a diameter of 5 mm was positioned near the center of the ROI. Within this circle, mean shear modulus values were automatically calculated (Fig. 1) [14, 21]. Furthermore, to minimize experimental errors, participants were advised not to engage in vigorous physical activity for two days leading up to the testing. The room temperature was maintained at 25° to minimize the effect of temperature on muscle elasticity [18].

To assess inter-rater reliability, all participants were independently measured on the same day by two radiologists with four years of experience in elastography and 40 h of training on shear modulus measurements of the muscles under study. The measurements were performed with a 5-min rest period between each measurement [21]. Each radiologist was blinded to the data of the other operator. After one week, a second measurement was conducted for assessing intra-rater reliability by both radiologists, without knowledge of the results from the first measurements [22, 23]. For each participant, three scans of the stiffness of each muscle (PM, MG, and LG) were performed by each radiologist, and their averages were used for statistical analysis.

### Statistical analysis

Using STATA SE14.2 software by the command “sampicc p_1_(0.9) number of replicates [4], CI level (95%), specified width (0.14)”, the sample size of 29 was calculated. IBM SPSS Statistics 26 was utilized for statistical analyses. The data were presented as mean ± SD. Their normality was evaluated using the Shapiro–Wilk test. A paired t-test was employed to compare the first and second measurements conducted by a single rater. The intra-rater (measurements at two time points, one week apart) and inter-rater (measurements by two operators) reliabilities of the shear modulus measurements for each muscle were evaluated using the intraclass correlation coefficient (ICC), employing a two-way mixed model and a 95% confidence interval. Additionally, assessment was performed using Bland–Altman plots and combo plots.

To evaluate the absolute reliability, we computed the standard error of measurement (SEM) using the formula: SEM = SD pooled × √ (1—ICC) [10, 24]. To identify clinically significant differences between 2 measurements, the minimal detectable change as (MDC = 1.96 × SEM × √2) and the coefficient of variation as (CV = [standard deviation/mean] × 100%) were computed [10, 24]. Statistical significance was established at a threshold of *P* < 0.05 for all measurements. ICC values < 0.5 are categorized as weak, ICC = 0.5—0.75 are medium, ICC = 0.75—0.9 are good, and ICC ˃ 0.9 are excellent [10, 24].

## Results

The study screened 54 individuals for participation. Nonetheless, only 30 individuals (22 women and 8 men) satisfied the eligibility requirements and joined the study. The age, weight, height and Body mass index (BMI) variables for the remaining 30 participants are presented in Table 1. The mean values of shear moduli for the PM, MG, and LG muscles between the two testers during the test and retest sessions showed no significant difference (Table 2).

**Table 1:** Participantsˈ demographics information (*N* = 30 individuals)

**Characteristic**	**Mean & SD**	**Min**	**Max**
**Age**	63.00 ± 6.71	50	70
**Weight**	70.83 ± 13.34	45	99
**Height**	1.63 ± 0.10	1.44	1.83
**BMI**	26.61 ± 4.28	17.63	33.30

*SD* standard deviation, *Min* minimum, *Max* maximum, *BMI* body mass index

**Table 2:** Descriptive statistics for Test–Retest Shear Modulus Measures of popliteus and medial and lateral gastrocnemius muscles

Muscle	First Examiner						Second Examiner					
	Test 1 (kPa)		Test 2 (kPa)				Test 1 (kPa)		Test 2 (kPa)			
	**Mean**	**SD**	**Mean**	**SD**	**P**	**E. size** **(CI:95%)**	**Mean**	**SD**	**Mean**	**SD**	**P**	**E. size** **(CI:95%)**
**PM**	12.75	3.00	12.61	2.89	.475	-.13 (-.49; .23)	11.66	3.13	11.62	3.04	.802	-.05 (-.40; .31)
**GM**	13.72	2.96	13.43	2.54	.454	-.14 (-.50; .22)	12.81	2.91	12.87	2.89	.799	.05 (-.31; .41)
**GL**	14.13	3.34	14.26	3.16	.694	.07 (-.29; .43)	13.17	3.45	13.30	3.14	.648	.08 (-.28; .44)

*PM* popliteus muscle, *GM* medial gastrocnemius, *GL* lateral gastrocnemius, *SD* standard deviation, *kPa* kilopascal, *P* p value, *E. size* effect size, *CI* confidence interval

The mean shear moduli of the PM, MG, and LG muscles in the test session were 12.75, 13.72, and 14.13 kilopascals (kPa) for tester 1, and 11.66, 12.81, and 13.17 kPa for tester 2. In the retest session, the mean values of these parameters were 12.61, 13.43, and 14.26 kPa for tester 1, and 11.62, 12.87, and 13.30 kPa for tester 2.

The values of MDC, SEM, CV and ICC for intra- and inter-rater reliability of the mean shear moduli of PM, MG, and LG muscles are displayed in Table 3. The ICC values indicated excellent reliability for intra-rater measurements in the PM, MG, and LG muscles (ICC for tester 1: 0.968, 0.922, and 0.912, respectively; ICC for tester 2: 0.986, 0.955, and 0.942, respectively). Additionally, the SEM values were calculated as 0.36—0.96, MDC values as 0.99—2.62, and CV values as 20.08%—26.29%.

**Table 3:** Intra- and Inter-rater Reliabilities of SWE for Mean Shear Modulus of popliteus and medial and lateral gastrocnemius muscles

**Reliability**		**Muscle**	**ICC (95%CI)**	**SEM**	**MDC**	**CV (%)**
**Intra-rater** **(Inter-day)**	First Examiner	PM	0.968 (0.932—0.985)	0.52	1.43	23.03
		GM	0.922 (0.835—0.963)	0.84	2.29	20.08
		GL	0.912 (0.815—0.958)	0.96	2.62	22.69
	Second Examiner	PM	0.986 (0.971—0.993)	0.36	0.99	26.29
		GM	0.955 (0.905—0.979)	0.61	1.68	22.43
		GL	0.942 (0.878—0.972)	0.79	2.16	24.70
**Inter-rater**	Test	PM	0.956 (0.908—0.979)	0.65	1.78	25.33
		GM	0.905 (0.801—0.955)	0.91	2.49	22.16
		GL	0.927 (0.848—0.969)	0.92	2.52	24.91
	Retest	PM	0.932 (0.858—0.968)	0.78	2.13	24.59
		GM	0.766 (0.509—0.889)	1.43	3.94	22.59
		GL	0.875 (0.737—0.940)	1.12	3.07	22.93

*SWE* Shear Wave Elastography, *PM* popliteus muscle, *GM* medial gastrocnemius, *GL* lateral gastrocnemius, *ICC* intraclass correlation coefficient, *SEM* standard error of measurement, *MDC* minimal detectable change, *CV* coefficient of variation

Furthermore, the ICC values indicated good to excellent reliability for inter-rater measurements in the PM, MG, and LG muscles. The inter-rater reliability ICC values for the mentioned muscles were reported as (0.932—0.956), (0.766—0.905), and (0.875—0.927) respectively. Moreover, the SEM values were calculated as 0.65—1.43, MDC values as 1.78—3.94, and CV values as 22.16%—25.33%.

**Fig. 2:**
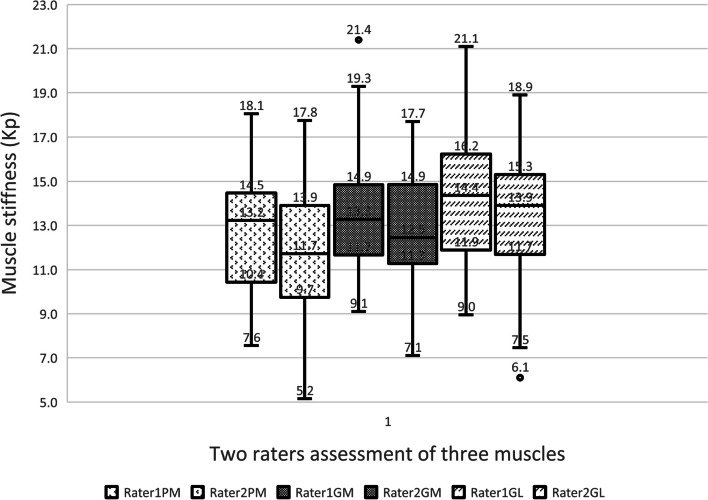
A boxplot illustrates the median muscle stiffness assessed by each rater for three muscles (PM (popliteus muscles), GM (gastrocnemius, medial head), and GL (gastrocnemius, lateral head), as well as the minimum and maximum stiffness (range) measured by each rater, including any outliers

In summary, the intra-rater reliability (ICC = 0.912—0.986) and inter-rater reliability (ICC = 0.766—0.956) for the shear moduli of the PM, MG, and LG muscles were good to excellent. The SEM was (0.36—1.43 kPa), MDC was (0.99—3.94 kPa), and CV was (20.08% to 26.29%). Fig. 2 illustrates the overlap in assessments in two raters and highlight the proximity of their evaluations.

**Fig. 3:**
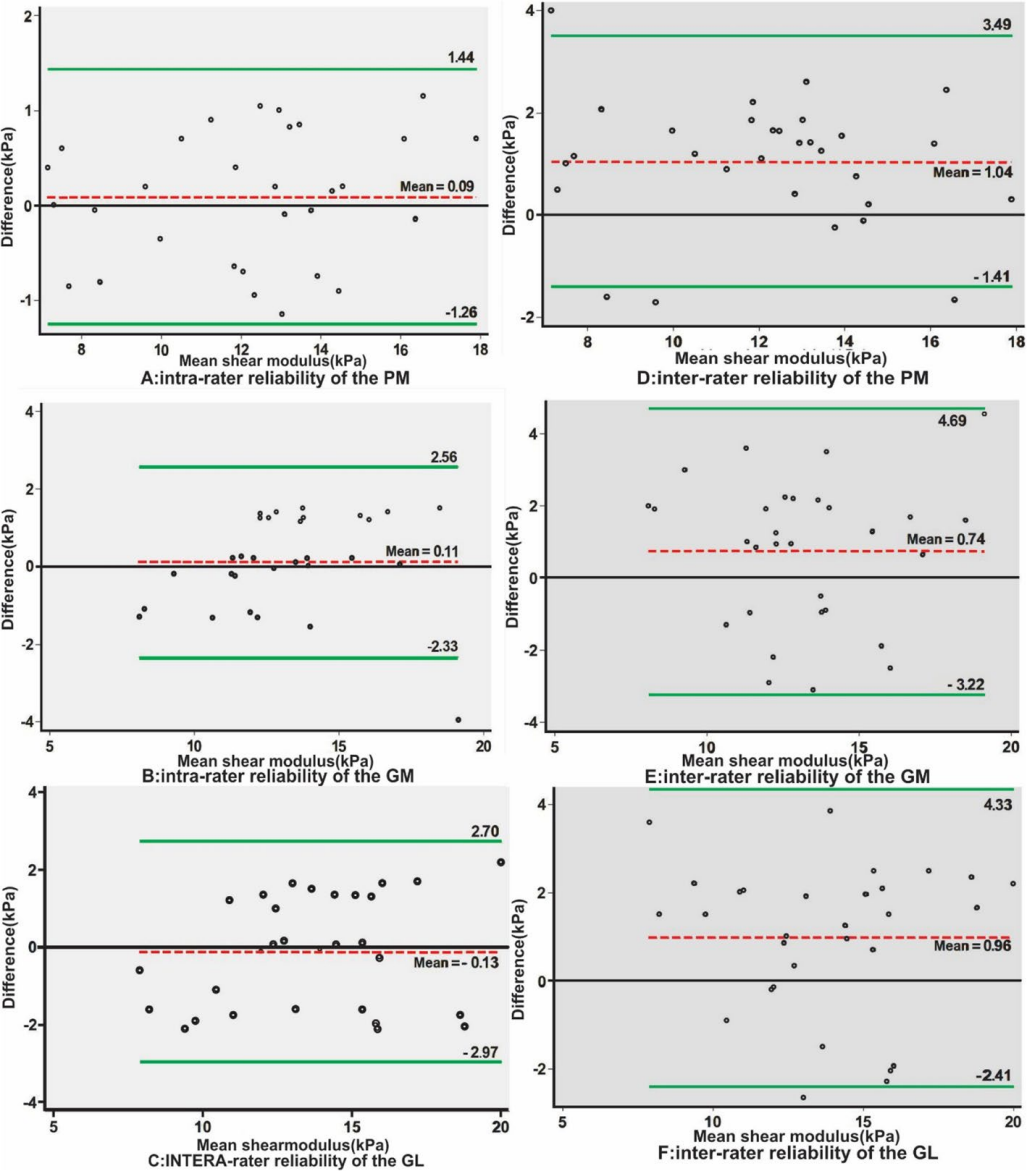
Bland–Altman plots illustrating intra- and inter-rater reliabilities for the shear moduli of popliteus, medial and lateral gastrocnemius muscles. The difference in shear moduli between test and retest session is plotted against the mean shear modulus of each participant for these muscles (**A**, **B**, **C**). The difference in shear modulus between the two testers is plotted against the mean shear modulus of each participant for popliteus, medial and lateral gastrocnemius muscles (**D**, **E**, **F**). In the images, the dotted line represents the mean difference, and the solid lines above and below indicate the limits of agreement

The Bland–Altman plots illustrating intra-rater reliability values for the PM, MG, and LG muscles are shown in Fig. 3A, B, and C, respectively. The mean differences were 0.09, 0.11, and 0.13 kPa, and the 95% limits of agreement were 1.26 to 1.44 kPa, 2.33 to 2.56 kPa, and 2.97 to 2.70 kPa, respectively. The inter-rater reliability values are shown in Fig. 3D, E, and F. The mean differences were 1.04, 0.74, and 0.96 kPa, and the 95% limits of agreement were 1.41 to 3.49 kPa, 3.22 to 4.69 kPa, and 2.41 to 4.33 kPa, respectively.

## Discussion

To date, Shear modulus measurement reliability using SWE in the PM, MG, and LG muscles has not been reported in participants with knee osteoarthritis who have myofascial trigger points in these muscles. In the current study, the measurements performed using SWE demonstrate good inter-rater reliability (ICC = 0.766—0.956) and excellent intra-rater reliability (ICC > 0.912). These findings, along with relatively low values of MDC and SEM, support the accuracy of measurements in this method our results align with previous studies concerning muscle shear modulus measurementsʹ reliability using SWE [22]. Despite its excellent reliability, the coefficient of variation was relatively high (CV = 20.08 to 26.29%). However, it is compatible with the findings of Jipping Zhu et al. [10] (CV = 21.5% to 27.69% cv) and Joseph Kelly's study (CV = 7% to 31%) [9].

Nakamura et al. examined the intra-rater and inter-rater reliability of the shear modulus in the medial head of the MG muscle in 10 young and healthy individuals, reporting strong reliability (ICC = 0.830—0.985) [21]. In the study conducted by Jipping Zhou et al., 21 healthy males participated, and the fascial stiffness of the MG and LG muscles was examined using SWE. They exhibited strong intra- and inter-rater reliability (ICC = 0.846—0.961) [10].

In the present study, the mean shear modulus of the PM muscle was higher (12.16 ± 3.02) compared to the study by Yagi et al. [14] (PM: 4.8 ± 10) and the study by Nakamura et al. [25] (GM: 7.8 ± 3.4 and GL: 8.5 ± 2.1). However, our subjects were individuals with knee osteoarthritis accompanied by trigger points. Therefore, it can be concluded that individuals with knee osteoarthritis accompanied by myofascial trigger points may exhibit higher muscle stiffness compared to healthy individuals. This increase in muscle stiffness could be attributed to the presence of myofascial trigger points in addition to knee osteoarthritis. Contrary to the findings of Nakamura et al., the study by Sobolewski et al. demonstrated that stiffness tends to be higher in older individuals [25] which may explain higher stiffness values in our study.

In the Bland–Altman plots analysis, in the intra-rater reliability values show that the upper and lower limits of the means have high agreement (-1.26 to 1.44, -2.33 to 2.56 and -2.97 to 2.70) and the mean difference is very small (0.09–0.13), suggesting excellent intra-rater reliability. On the other hand, in the inter-rater reliability plots, the agreement between the upper and lower limits of the means has slightly decreased (-1.41 to 3.49, -3.22 to 4.69 and -2.41 to 4.33) and the mean difference have slightly increased (0.74–1.04), indicating that intra-rater reliability surpasses inter-rater reliability, in line with the findings reported in the study by Jipping Zhou [10].

The study conducted by Mahyar Salavati et al. on 32 trapezius muscles with myofascial trigger points demonstrated that in muscle stiffness measurements, intra-rater reliability (ICC = 0.75—0.81) was higher than inter-rater reliability (ICC = 0.7—0.75) [15]. Minsoo Jeon et al. examined gastrocnemius muscle stiffness in 15 healthy individuals using ultrasound elastography. They reported strong intra-rater reliability (ICC = 0.897—0.976) and good inter-rater reliability (ICC = 0.891) [22]. The finding from these studies is in accordance with the outcomes of our own study.

Finally, we observed a relatively high CV how much data varies compare to the average. It helps compare variations in different datasets. On the other hand, ICC checks how consistent ratings are for the same subject compared to variations across all ratings and subjects. In our study high CV values could be impacted by individual differences among subjects [26, 27].

### Future directions and clinical implications

Our findings demonstrated that SWE is a reliable tool for objectively measuring calf muscles stiffness in individuals with knee osteoarthritis accompanied by trigger points. Its affordability and accessibility render it suitable for clinical settings to objectively evaluate intervention effects. Furthermore, future studies should explore the potential of the SWE in diagnosing other musculoskeletal disorders.

## Limitations

Previous studies have highlighted the effects of sex on muscle stiffness [19], indicating higher stiffness in males compared to females. As we did not assess the influence of sex in our study, it might have impacted our results, potentially demonstrating a greater coefficient of variation. Additionally, a limitation of our study could stem from the older age of our sample size (50–70) which limits the generalizability of our finding to younger age groups, considering aging tends to increase muscle stiffness [25, 26]. Lastly, researchers should take into account that the generalizability of our study is confined to the sites were assessed.

## Conclusion

The measurement of muscle stiffness using SWE in the PM, MG, and LG muscles of individuals with knee osteoarthritis accompanied by myofascial trigger points demonstrates good to excellent intra-rater and inter-rater reliability. Therefore, when assessing the stiffness of the PM, MG, and LG muscles, utilizing the standardized points and probe orientation suggested in this study may be helpful in identifying myofascial trigger points and evaluating pre- and post-treatment changes in these muscles, particularly in individuals with knee osteoarthritis.

## Data Availability

The datasets used and/or analyzed during the current study are available from the corresponding author on reasonable request.

## Abbreviations

SWE: Shear Wave Elastography
PM: Popliteus Muscle
MG: Medial Gastrocnemius
LG: Lateral Gastrocnemius
ROI: Region of Interest
SEM: Standard Error of Measurement
MDC: Minimal Detectable Change
CV: Coefficient of Variation
SD: Standard Deviation
ICC: Intraclass Correlation Coefficient
BMI: Body Mass Index
E. size: Effect size
CI: Confidence interval
